# F-actin reorganization by V-ATPase inhibition in prostate cancer

**DOI:** 10.1242/bio.028837

**Published:** 2017-10-16

**Authors:** Yamhilette Licon-Munoz, Vera Michel, Colleen A. Fordyce, Karlett J. Parra

**Affiliations:** Department of Biochemistry and Molecular Biology, School of Medicine, University of New Mexico, Albuquerque, New Mexico 87131, USA

**Keywords:** Vacuolar H^+^-ATPase proton pump, Bafilomycin A, Concanamycin A, Endo-lysosomal pH, F-actin, Prostate cancer

## Abstract

The vacuolar ATPase (V-ATPase) proton pump sustains cellular pH homeostasis, and its inhibition triggers numerous stress responses. However, the cellular mechanisms involved remain largely elusive in cancer cells. We studied V-ATPase in the prostate cancer (PCa) cell line PC-3, which has characteristics of highly metastatic PCa. V-ATPase inhibitors impaired endo-lysosomal pH, vesicle trafficking, migration, and invasion. V-ATPase accrual in the Golgi and recycling endosomes suggests that traffic of internalized membrane vesicles back to the plasma membrane was particularly impaired. Directed movement provoked co-localization of V-ATPase containing vesicles with F-actin near the leading edge of migrating cells. V-ATPase inhibition prompted prominent F-actin cytoskeleton reorganization. Filopodial projections were reduced, which related to reduced migration velocity. F-actin formed novel cytoplasmic rings. F-actin rings increased with extended exposure to sublethal concentrations of V-ATPase inhibitors, from 24 to 48 h, as the amount of alkalinized endo-lysosomal vesicles increased. Studies with chloroquine indicated that F-actin rings formation was pH-dependent. We hypothesize that these novel F-actin rings assemble to overcome widespread traffic defects caused by V-ATPase inhibition, similar to F-actin rings on the surface of exocytic organelles.

## Abstract (precis)

**Summary:** V-ATPase activates multiple stress responses. In prostate cancer, sub-lethal concentrations of V-ATPase inhibitors trigger widespread traffic defects. F-actin assembles into rings that mimic those seen during regulated exocytosis.

## INTRODUCTION

Membrane compartmentalization allows eukaryotic cells to organize functions by grouping them in membrane-bound vesicles. Compartmentalization is maintained through vesicle transport (Cho and Stahelin, 2005; Miaczynska et al., 2004; Pfeffer, 2003) which relies upon differential pH gradients (Casey et al., 2010; Paroutis et al., 2004). Both processes require vacuolar type H^+^-ATPase [vacuolar ATPase (V-ATPase)] proton pumps (Paroutis et al., 2004; Sobota et al., 2009). V-ATPase is a multisubunit protein complex that comprises two functional domains: V_1_ and V_o_ (Forgac, 2007; Toei et al., 2010). The catalytic domain (V_1_) hydrolyzes cytosolic ATP, which powers proton transport via the membrane-embedded domain (V_o_). Active transport of protons by V-ATPase acidifies endosomes, lysosomes, Golgi-derived vesicles, clathrin-coated vesicles, and secretory vesicles (Forgac, 2007; Hinton et al., 2009; Toei et al., 2010).

V-ATPase proton transport also generates a membrane potential that is necessary to activate secondary transport systems (Forgac, 2007). V-ATPase-dependent organelle acidification and membrane energization are important in several cellular processes, particularly those that rely on membrane trafficking. Receptor- or clathrin-mediated endocytosis, endosomal vesicle budding and cargo distribution, protein maturation, and lysosome biogenesis require functional V-ATPases (Marshansky and Futai, 2008). In addition to intracellular V-ATPases, certain cells specialized for active proton secretion also express V-ATPase at the plasma membrane. In clear cells of the epididymis (Pietrement et al., 2006), alpha-intercalated cells of the kidney (Wagner, 2008), and osteoclasts (Toyomura et al., 2003), plasmalemmal V-ATPase acidifies the extracellular milieu which is critical for sperm maturation, urine acidification, and bone resorption, respectively.

In cancer cells, plasma membrane-associated V-ATPases have been largely linked to cancer migration and invasive phenotypes (Capecci and Forgac, 2013; Cotter et al., 2015; Michel et al., 2013; Montcourrier et al., 1997). Cancer tumor cell lines with high metastatic potential express more V-ATPase pumps at the plasma membrane than less aggressive cell lines (Cotter et al., 2015; Hinton et al., 2009; Michel et al., 2013; Smith et al., 2016). Extracellular acidification by V-ATPase activates cathepsin (Hinton et al., 2009; Kubota and Seyama, 2000; Sennoune et al., 2004), which is required for cell motility and invasion.

Their role in metastasis and cell death makes V-ATPase proton pumps attractive targets to combat cancer. V-ATPase is involved in angiogenesis. The pigment epithelium-derived factor, a potent inhibitor of angiogenesis, was shown to down-regulate expression of V-ATPase at the plasma membrane in the lung metastatic CL1 cell line (Rath et al., 2014; Rojas et al., 2006; Sennoune et al., 2014). In addition, the uptake of chemotherapeutic drugs is sensitive to pH alterations. V-ATPase facilitates sequestration of chemotherapeutic agents in acidic compartments, which contributes to drug resistance (Gerweck et al., 2006; Milito et al., 2007; von Schwarzenberg et al., 2014; You et al., 2009). Loss of V-ATPase function promotes apoptosis by caspase-dependent and -independent mechanisms in several cancer cell lines (Aiko et al., 2002; Ishisaki et al., 1999; McHenry et al., 2010; Morimura et al., 2008; Nakashima et al., 2003; Sasazawa et al., 2009; Wang et al., 2008).

V-ATPase activity is linked to several cellular events in prostate cancer (PCa) cells. V-ATPase inhibitors cause PCa cell apoptosis and cell cycle arrest (Wang et al., 2008). V-ATPase is crucial for normal prostate-specific antigen (PSA) physiology. V-ATPase inhibitors suppress PSA expression, alter PSA intracellular distribution, and reduce PSA secretion (Michel et al., 2013). It has been reported that PCa V-ATPase activity is regulated by the tumor metastasis suppressor gene 1 (Xu et al., 2014; Yu et al., 2013) and the pigment epithelium-derived factor (Sennoune et al., 2014). In highly aggressive PCa cell lines such as PC-3, V-ATPase is required for delivery of the membrane-bound matrix metalloproteinase MMP-14 to the plasma membrane, as well as cell growth and invasiveness (Smith et al., 2016; Wang et al., 2008).

We used the prostate adenocarcinoma PC-3 cell line in this study. PC-3 is derived from bone metastasis of a human prostate carcinoma and possess many of the characteristics of a highly malignant neoplasm (Kaighn et al., 1979; Sobel and Sadar, 2005a,b). We studied the downstream physiological consequences of inhibiting V-ATPase in these PC-3 prostate carcinoma cells, from V-ATPase distribution, invasion, and migration to its effects on the organization of F-actin. We report that V-ATPase inhibition causes F-actin cytoskeleton reorganization in PC-3. It reduces or eliminates the filopodia projecting from the cell surface and provokes accumulation of F-actin ring structures. The F-actin rings differ from invadopodia, as the rings are depleted of vinculin, and invadopodia-associated processes such as vesicle trafficking, cell invasion, and migration are defective (Desai et al., 2008). While prior studies suggest that normal arrangement of filamentous actin is disrupted if V-ATPase is defective (Feng et al., 2014; Kazami et al., 2014; Zhang et al., 1998), V-ATPase-dependent F-actin ring assemblies have not been previously reported.

## RESULTS

Inhibition of pH regulation has been proposed as a therapeutic strategy in cancer cells (Meehan et al., 2017) because V-ATPase is involved in metastasis and is exploited by tumors to survive, proliferate, and resist therapy (Stransky et al., 2016). Development of chemotherapeutic V-ATPase inhibitors requires understanding the complexity of cellular processes deregulated upon V-ATPase inhibition. However, the mechanisms that regulate these processes remain mainly elusive.

### PC-3 cells predominantly express V-ATPase V_o_a2 and V_o_a3 subunit isoforms

**Fig. 1.**
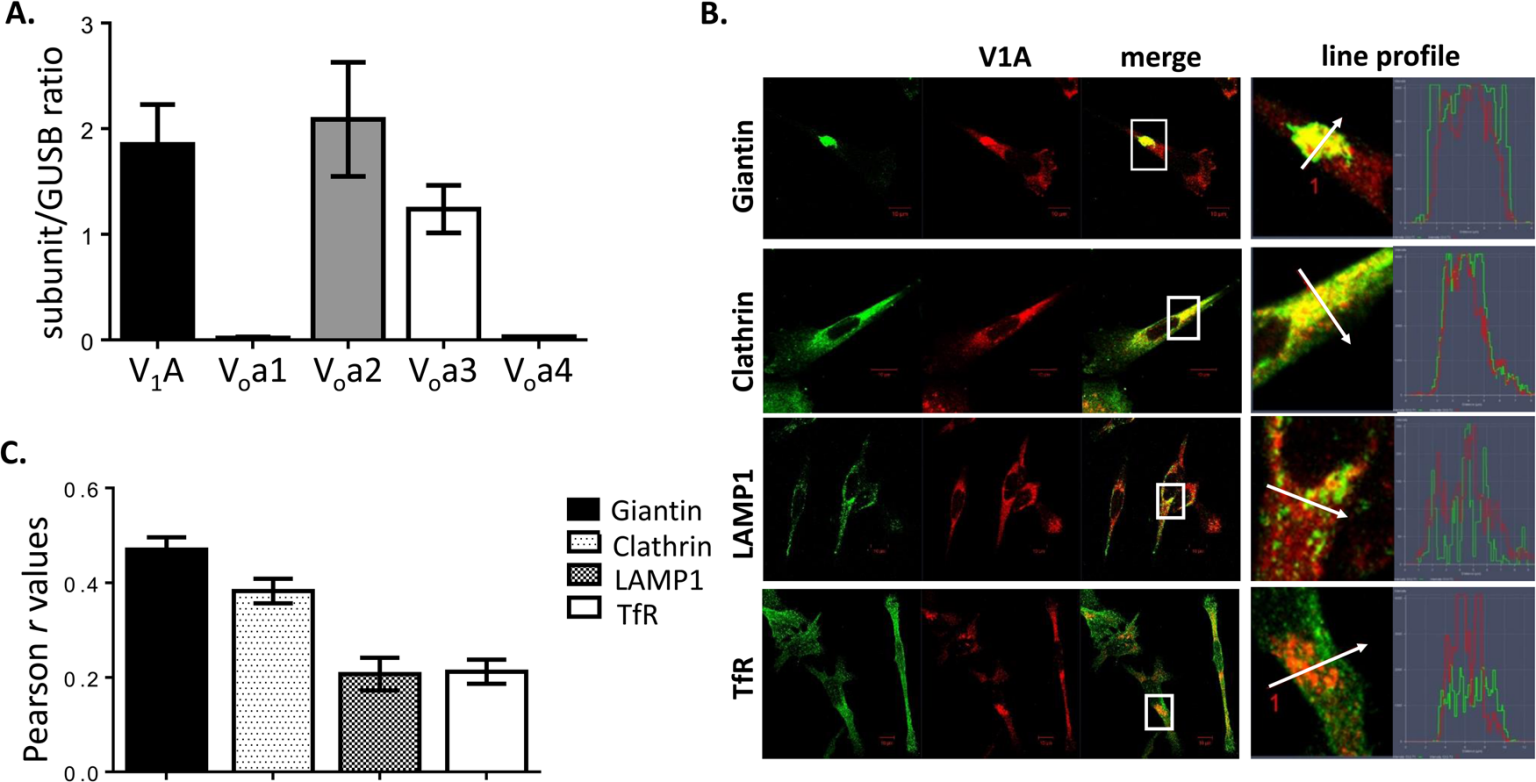
**V-ATPase expression and distribution in PC-3 cells.** (A) V-ATPase subunit mRNA (V_1_A, V_o_a1, V_o_a2, V_o_a3, and V_o_a4) was quantified by qRT-PCR and normalized to β-glucuronidase (GUSB) in PC-3 cells. Data are expressed as mean±s.e.m. (B) The cells were immunostained with antibodies against the V-ATPase subunit V_1_A and markers of the Golgi compartment (giantin), clathrin-coated vesicles (clathrin), lysosomes (LAMP1) and recycling endosomes (transferrin receptor, TfR). Co-localization was analyzed using confocal microscopy determining a line profile of fluorescence intensity. Arrow shows line profile *x*-axis. Scale bar: 10 µm. (C) Pearson *r* values were obtained to characterize the degree of overlap between V_1_A signal and either giantin, clathrin, LAMP1 or transferrin receptor (TfR). Data (*r* values) are expressed as mean±s.e.m. *n*=50 cells.

Human cells express four isoforms of the V-ATPase V_o_ subunit a (V_o_a1, V_o_a2, V_o_a3, and V_o_a4) (Forgac, 2007; Marshansky and Futai, 2008) that target V-ATPase to different cellular membranes (Forgac, 2007; Marshansky and Futai, 2008; Saw et al., 2011). To determine the V_o_ subunit-a isoform preferentially expressed in PC-3 cells, we used qRT-PCR to measure relative expression of these four V_o_a isoforms. The subunit V_1_A is assembled in every V-ATPase complex, regardless of the membrane and cell type. Thus, the subunit V_1_A was monitored as a means of detecting all V-ATPase complexes in PC-3 cells. PC-3 cells expressed comparable amounts of V_o_a2 and V_o_a3 but did not have detectable levels of V_o_a1 or V_o_a4 (Fig. 1A). These results suggest that V-ATPase complexes containing the V_o_a2 and V_o_a3 isoforms are predominant in PC-3 cells.

### V-ATPase is distributed in the Golgi compartment, endosomes and lysosomes

We used immunofluorescence confocal microscopy to analyze the cellular distribution of V-ATPase pumps in PC-3 cells, using antibodies against the V-ATPase subunit V_1_A (Michel et al., 2013) and markers for various compartments of the endomembrane system. As anticipated, V-ATPase was present in the Golgi compartment (giantin), clathrin-coated vesicles (clathrin), lysosomes (LAMP1), and recycling endosomes (transferrin receptor, TfR) (Fig. 1B). However, the Pearson correlation coefficient (Pearson's *r*) that measures the linear correlation between two variables was greater for giantin and clathrin, indicating that there was a higher degree of co-localization (∼two-fold) of V_1_A with giantin and clathrin (Pearson *r*=0.46±0.02 and *r*=0.38±0.02, respectively) than with LAMP1 or TfR (Pearson *r*=0.20±0.03 and *r*=0.21±0.02, respectively) (Fig. 1C). Thus, in PC-3 cells, V-ATPases are primarily located in the Golgi compartment and clathrin-coated vesicles with fewer detectable V-ATPases in the lysosomes and recycling endosomes.

### V-ATPase inhibitors disturb organelle acidification and endomembrane trafficking

**Fig. 2.**
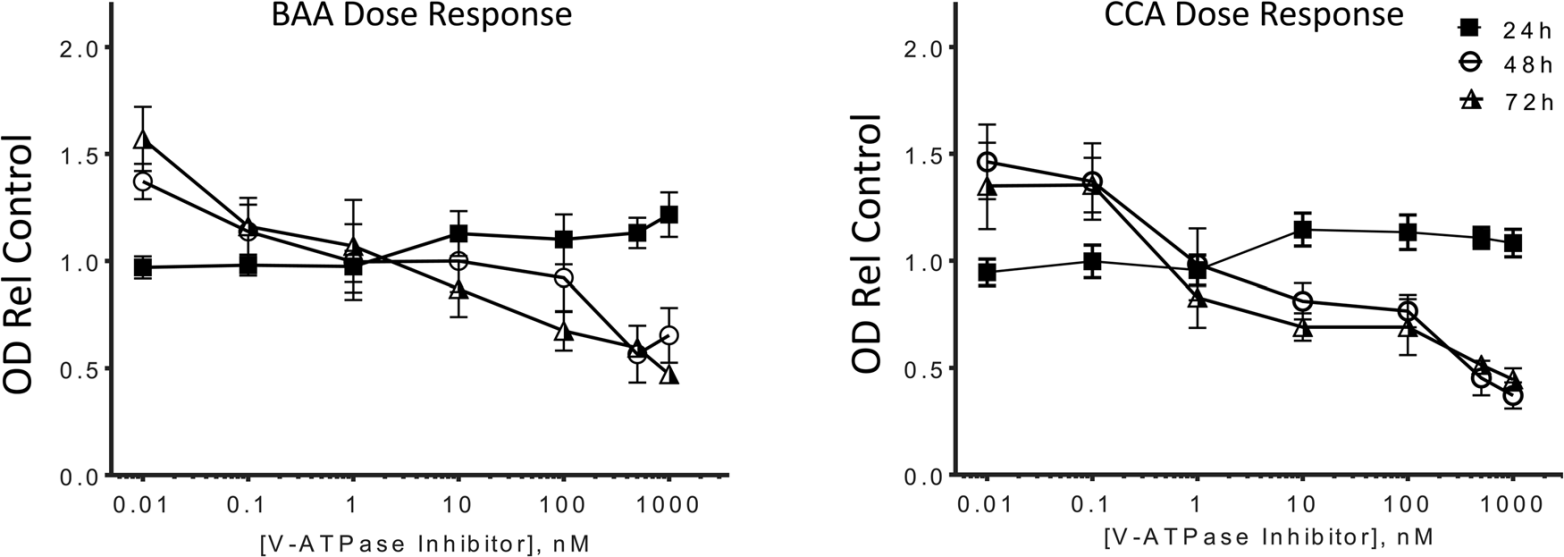
**V-ATPase inhibition diminishes PC-3 cell survival in a dose-dependent manner.** MTT viability of PC-3 cells treated for 24, 48, and 72 h with the indicated concentrations of bafilomycin A (BAA) or concanamycin A (CCA) was assessed. Data are expressed as OD relative to the vehicle-treated control. Mean±s.e.m.; *n*=3.

We treated PC-3 cells with the plecomacrolide antibiotics bafilomycin A (BAA) and concanamycin A (CCA), two highly potent and specific V-ATPase inhibitors that bind to the proteolipid subunit c in the V_o_ domain, which is directly involved in proton transport (Bowman et al., 1988; Dröse and Altendorf, 1997; Huss and Wieczorek, 2009). These inhibitors are frequently used to study V-ATPase in a variety of cell types (Huss and Wieczorek, 2009). However, prolonged exposure to these plecomacrolides can result in cell death (Dröse and Altendorf, 1997). Therefore, we first assessed PC-3 cell viability. We measured metabolic activity as the reduction of Tetrazolium MTT by NAD(P)H-dependent oxidoreductases in viable PC-3 cells exposed to 0.01-1000 µM BAA or CCA at 24, 48, and 72 h. The V-ATPase inhibitors BAA and CCA reduced cell viability in a dose- and time-dependent manner (Fig. 2). The fraction of living cells relative to control untreated cells decreased upon exposure to concentrations above 10 nM for the 48 and 72 h treatments, but not at 24 h.

**Fig. 3.**
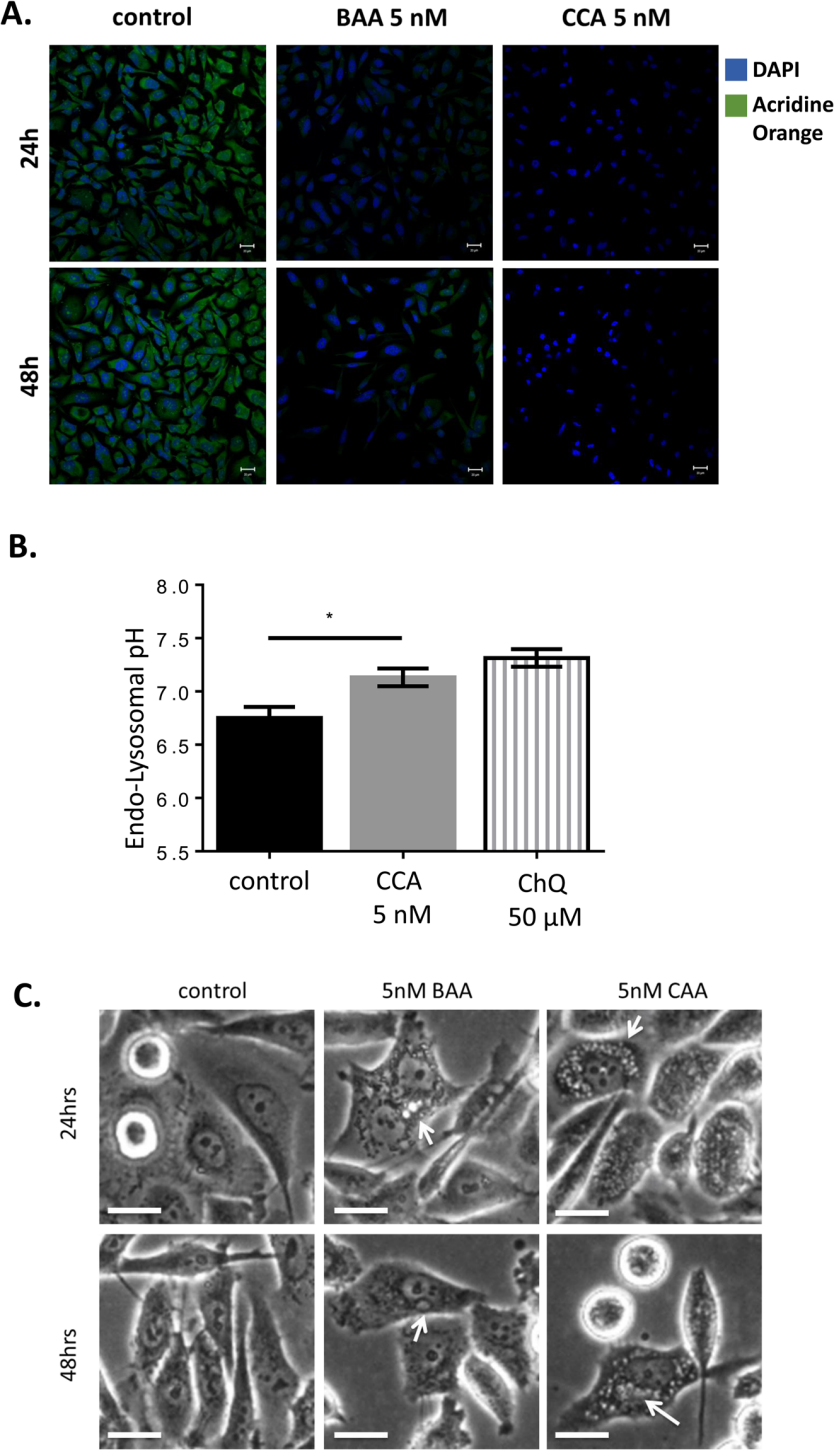
**V-ATPase inhibition disturbs organelle acidification and triggers intracellular vesicles accumulation in PC-3 cells.** (A) PC-3 cells were treated with 0.005% DMSO (control), BAA (5 nM), or CCA (5 nM) for 24 h (top panel) and 48 h (bottom panel). Intracellular pH was qualitatively assed using the pH-sensitive dye Acridine Orange. Acridine Orange accumulates in acidic vesicles and emits fluorescence at low pH. Cells were stained with 1 µM Acridine Orange (green) for 30 min and analyzed using fluorescent confocal microscopy. DAPI (blue) was used as nuclear marker. The decrease or loss of green fluorescence indicates alteration in organelle acidification. Scale bar: 20 µm. (B) PC-3 cells were incubated with the pH-sensitive fluorescent dye HPTS for 24 h and then treated with DMSO 0.005% (control), 5 nM CCA, or 50 µM of chloroquine (ChQ) for 1 h. Cells were collected, washed and analyzed using a fluorometer. Endo-Lysosome pH was determined by comparing the fluorescence with an excitation ratio of 458/405 nm at a fixed emission of 515 nm to a standard curve generated using known pH buffers. Mean endosome and lysosome pH is shown ±s.e.m. from 3-5 experiments. *P<0.05; Mann-Whitney test. (C) Phase contrast images of PC-3 cells treated with vehicle control (DMSO 0.005% in media), 5 nM BAA, or 5 nM CCA for 24 h (top panel) and 48 h (bottom panel) are shown. Arrows indicate vesicle accumulation. Images were obtained with a Primo Vert microscope. Scale bar: 20 µm.

Although 5 nM concentrations of BAA and CCA did not significantly decrease cell viability at 24 and 48 h (Fig. 2), V-ATPase function was inhibited at this concentration. Intracellular organelle acidification was disrupted upon treatment with BAA and CCA (Fig. 3A), as measured by decreased accumulation of the weak lipophilic base Acridine Orange relative to control cells exposed to vehicle alone (DMSO). To quantify these observations, we measured the luminal pH of endosomes and lysosomes fluorometrically using the pH-sensitive fluorescent dye HPTS, which is trapped in acidic compartments via endocytosis (Overly et al., 1995). For these studies, PC-3 cells were exposed to the V-ATPase inhibitors for 1 h, because prolonged exposure blocks endocytosis (Marshansky and Futai, 2008). Treatment with 5 nM CCA (Fig. 3B) increased the pH of endosomes and lysosomes from pH 6.7 to pH 7.1 (*P*=0.02). Chloroquine (50 µM), which alkalinizes these intracellular compartments independent of V-ATPase pumps, raised the pH to 7.3. We concluded that V-ATPase proton transport was effectively blocked in the PC-3 cells exposed to V-ATPase inhibitors at concentrations as low as 5 nM.

**Fig. 4.**
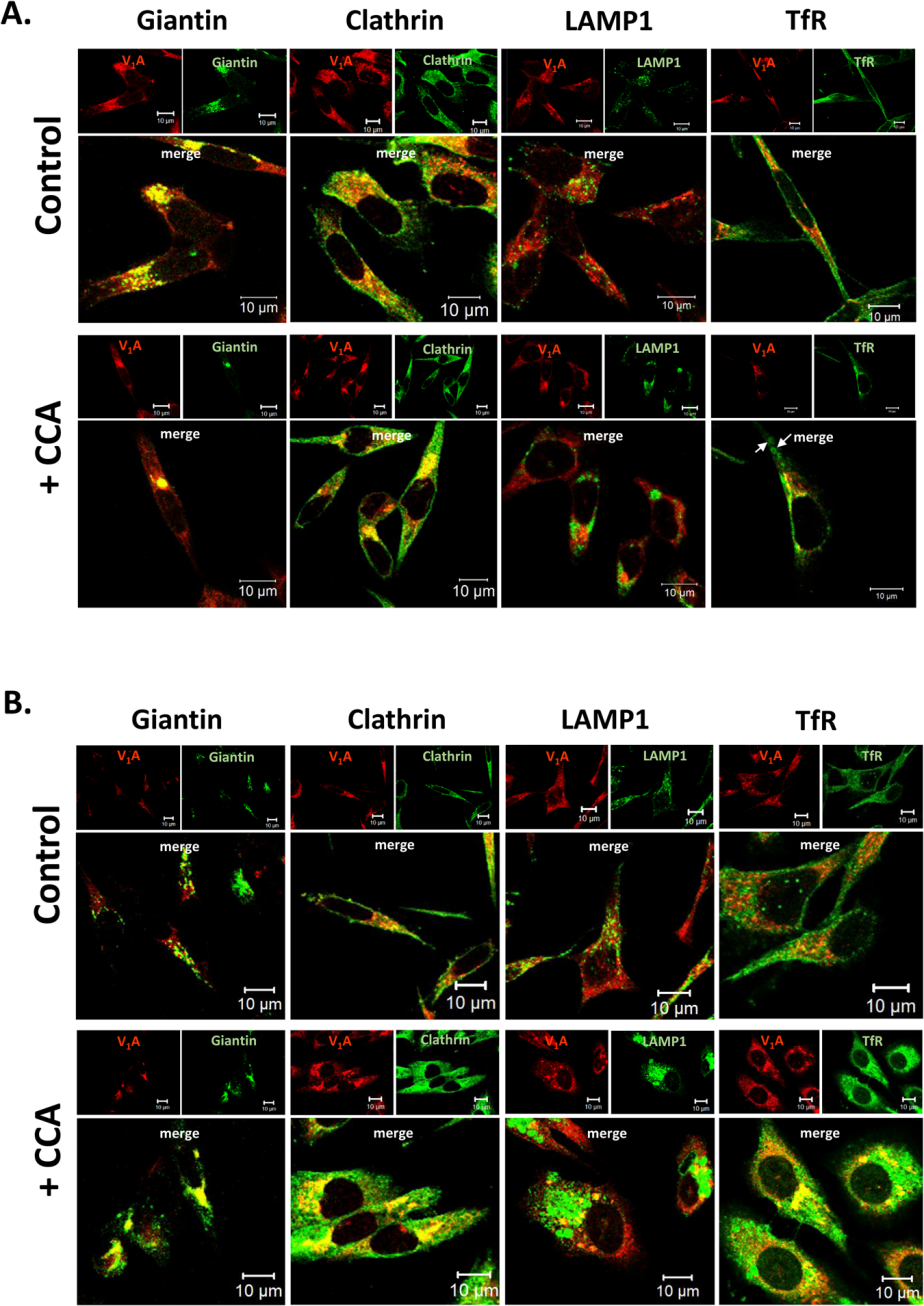
**V-ATPase inhibition leads to accumulation of lysosomes, clathrin-coated vesicles, and recycling endosomes.** (A) PC-3 cells were fixed after a 24 h incubation with vehicle control media (DMSO 0.005%) (top panel) or with 5 nM of V-ATPase inhibitor (+ CCA, botton panel). Cells were then co-immunostained with antibodies against the V-ATPase subunit V_1_A (red) and the indicated marker proteins (green). White arrows show ring structures positive for TfR. (B) PC-3 cells were fixed after 48 h incubation with the conditions described above. After treatment, accumulation of lysosomes, recycling endosomes, and chlatrin-coated vesicles was observed. Scale bars: 10 µm.

**Fig. 5.**
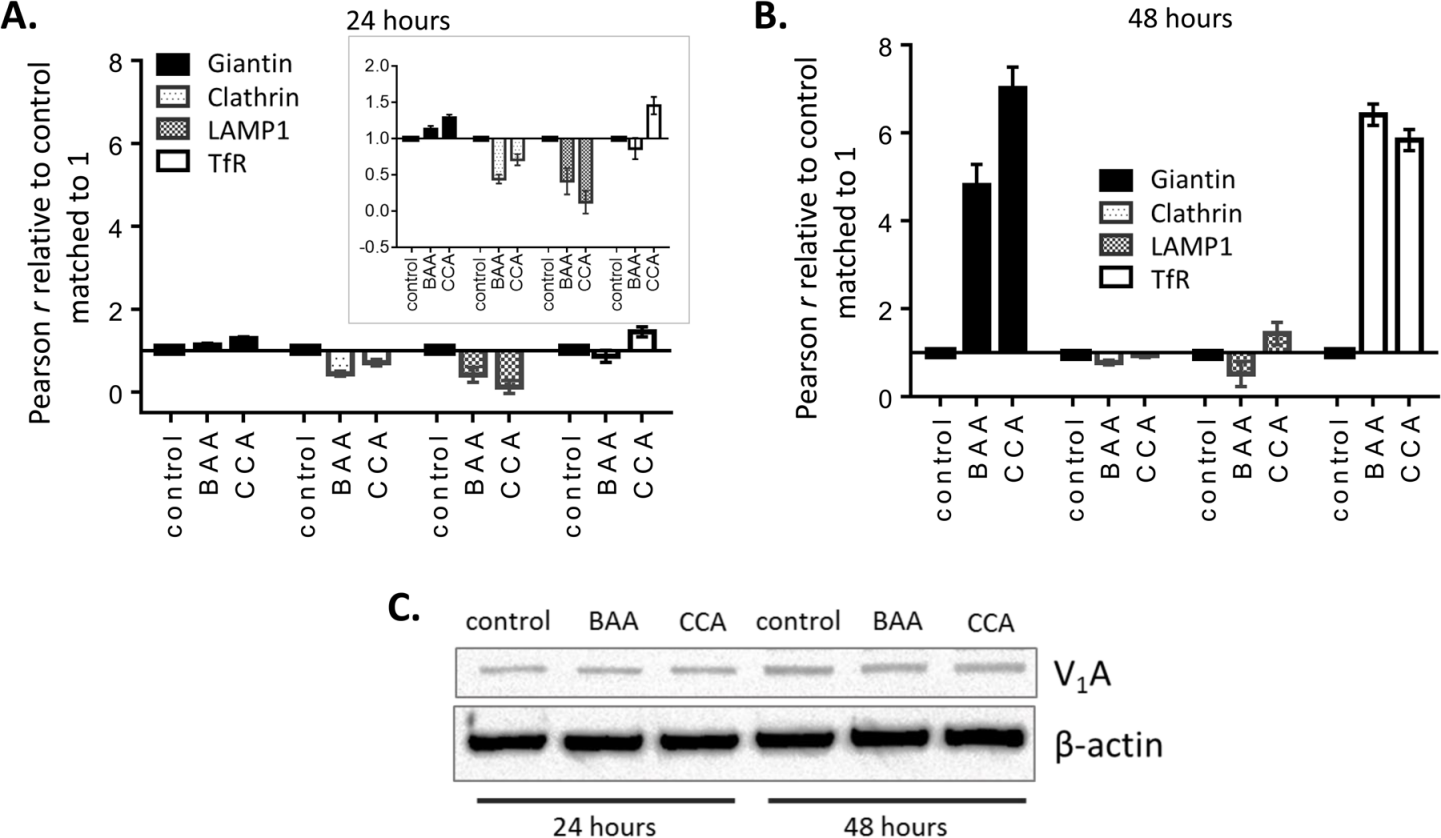
**Pearson analyses show V_1_A subunit accumulation in Golgi and endosomes.** (A) Pearson *r* values were obtained to characterize the degree of overlap between V_1_A signal and either giantin (Golgi), clathrin (clathrin-coated vesicles), LAMP1 (lysosomes) or transferrin receptor (endosomes, TfR). Confocal microscopy images were analyzed. Data are normalized as Pearson *r* score relative to control for each organelle marker; *n*=50 cells. The insert shows a zoom-in of the normalized Pearson *r* value scale. (B) Pearson *r* values were obtained as described for A, both in control conditions and after 48 h incubation with 5 nM BAA and CCA. Pearson *r* data are normalized relative control for each organelle marker; *n*=50 cells. Data are presented mean±s.e.m. in A and B. (C) PC-3 whole cell lysates were obtained after 24 or 48 h incubation with vehicle control media (DMSO 0.005%), 5 nM BAA, or 5 nM CCA. Western blots were used to monitor the V-ATPase subunit V_1_A (Michel et al., 2013) and β-actin (loading control).

Loss of pH gradients upon V-ATPase inhibition impairs membrane turnover and endocytic processes (Straud et al., 2010). Accordingly, BAA and CCA treatments increased the number and size of intracellular vesicles detected by phase contrast (Fig. 3C), which were further examined by confocal microscopy. Immunocytochemistry showed increased signals for clathrin (clathrin-coated vesicles), LAMP1 (lysosomes), transferrin receptor (recycling endosomes), and giantin (Golgi) following V-ATPase inhibition (Fig. 4). The lysosomes, clathrin-coated vesicles, and recycling endosomes were larger and more numerous after 48 h, as the respective markers (LAMP1, clathrin, and transferrin receptor) were increased after CCA exposure as compared to control (Fig. 4B). Pearson's *r* values increased five- to seven-fold recycling endosomes, indicating that V-ATPase was retained in these compartments (Fig.5A,B). In contrast, a modest decrease relative to controls was measured for LAMP-1 and clathrin-positive membranes at 24 and 48 h. Moreover, the level of Golgi-associated V-ATPase increased, as shown by its co-localization with giantin (Fig. 4). Significantly greater Pearson's *r* values in the Golgi at 48 h (Fig. 5B) indicate that vesicle trafficking from the Golgi compartment was also blocked. V-ATPase expression did not change, as the total level of V_1_A subunit detected in whole cell lysates by western blots was not different in PC-3 cells after treatment with BAA and CCA (Fig. 5C). Western blots showed that V-ATPase was stable, indicating that these Pearson's *r* value variations reflect vesicle traffic alterations. Together, these results indicate that V-ATPase function is required for V-ATPase to exit the Golgi and for distribution of V-ATPase to different cell membranes. They also indicate that V-ATPase activity is necessary for endocytic recycling of the transferrin receptor to the plasma membrane in the PC-3 cells.

### V-ATPase inhibition impairs *in vitro* motility and invasion

**Fig. 6.**
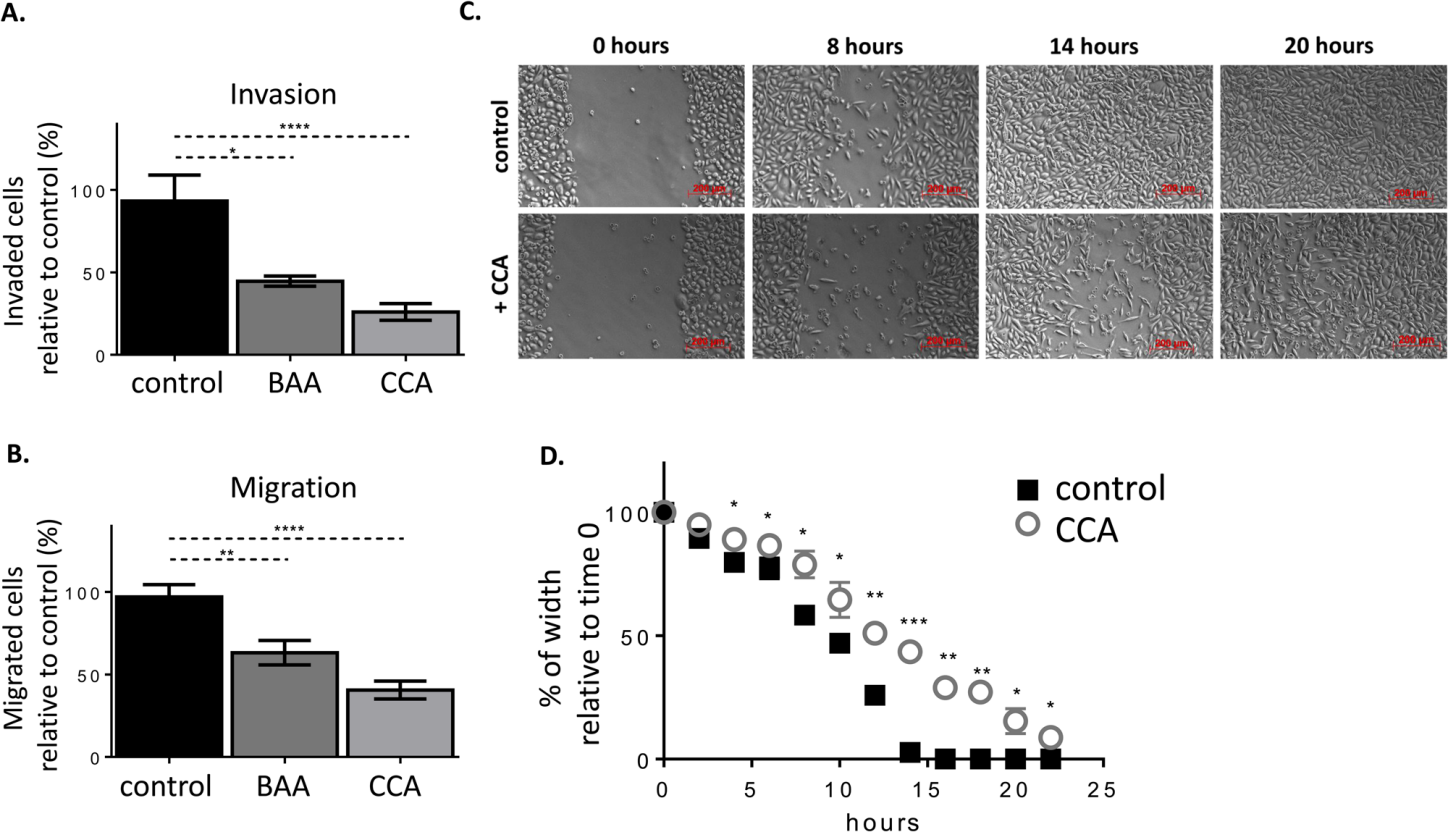
**PC-3 motility and invasion are impaired by V-ATPase inhibition.** (A) PC-3 cells were placed in matrigel-coated inserts (8 µm pores) in the absence (control) or presence of V-ATPase inhibitors (BAA or CAA at 5 nM) for 24 h. Fetal bovine serum (10% v/v) was used as a chemoattractant. Mean±s.e.m.; *P<0.05, ****P<0.0001, Mann-Whitney test, *n*=3 independent experiments. (B) PC-3 cells were placed in migratory inserts without Matrigel (8 µm pores) in the absence (control) or presence of V-ATPase inhibitors (BAA or CAA at 5 nM) for 24 h. Fetal bovine serum (10% v/v) was used as a chemoattractant. Mean±s.e.m.; **P<0.01, ****P<0.0001, Mann-Whitney test, *n*=3 independent experiments. (C) PC-3 cells were grown in a confluent monolayer and then a scratch was made to create a ‘wound’ and induce motility in the absence (DMSO 0.005%, top panel) or presence of 5 nM CCA (+CCA, bottom panel). Representative pictures at 0, 8, 14, and 20 h after the scratch were taken are shown. Images were obtained with an AxioVision 4.8 microscope. Scale bar: 200 µm. (D) PC-3 cells were imaged every 2 h and the scratch width (µm) values were expressed as % of width relative to time 0. Mean±s.e.m.; *P<0.05, **P<0.01, ***P<0.0001, Mann-Whitney test, *n*=3 repetitions. Comparable results were obtained after BAA exposure (not shown).

The resemblance of PC-3 to advanced PCa tumor cells with high metastatic potential is illustrated by the high motility and invasive phenotype of the cells (Overly et al., 1995; Sobel and Sadar, 2005a,b; Straud et al., 2010). These phenotypes were very sensitive to V-ATPase inhibition. Treatment with V-ATPase inhibitors significantly decreased *in vitro* invasion and migration by about 50% or more (Fig. 6A,B). Independent measurements using a wound-healing assay also showed V-ATPase-dependent inhibition of cell motility. When a confluent monolayer of cells treated with CCA was ‘wounded’ by scratching, the cells exhibited a significant delay in closing the wound width relative to vehicle-treated cells (DMSO). The time it took to close the wound was 1.6-fold longer for CCA- treated cells (23 h) than untreated cells (14 h) (Fig. 6C,D). Thus, PCa V-ATPase is likely intertwined with disease invasiveness, as inhibition of V-ATPase activity reduces PC-3 cell migration.

### Directed cell motility provokes redistribution of V-ATPase-containing vesicles

**Fig. 7.**
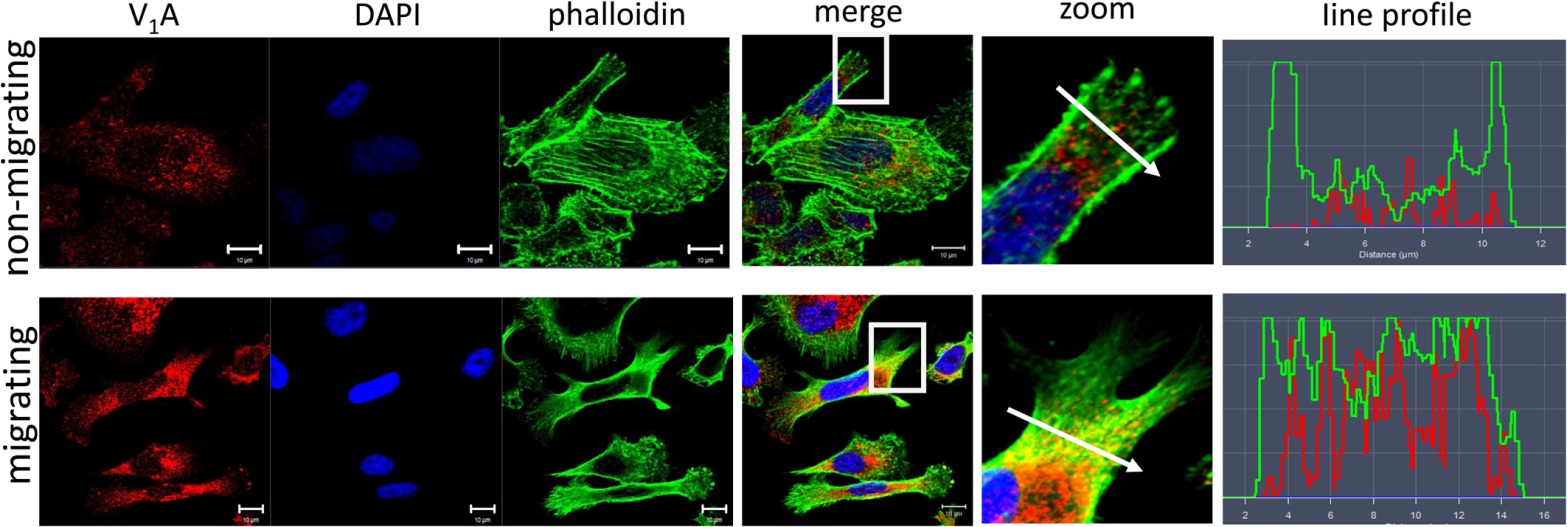
**V-ATPase is found in vesicles near the leading edge in migrating PC-3 cells.** PC-3 monolayers in control media were wounded as previously described for Fig. 6. Cells were fixed after 4 h. Representative non-migrating cells farthest from the wound (top) and migrating cells that had crawled to the middle of the wound (bottom) are shown. The distribution of V-ATPase subunit V_1_A (red) and phalloidin (F-actin, green) were visualized by confocal microscopy. DAPI (blue) was used as nuclear marker. Scale bar: 10 µm. Co-localization was analyzed by determining a line profile of fluorescent intensity as described for Fig. 1.

In several tumor cell lines, including PC-3, extracellular acidification by plasmalemmal V-ATPase was shown to contribute to *in vitro* invasion and migration (Cotter et al., 2015; Forgac, 2007; Hinton et al., 2009; Michel et al., 2013; Smith et al., 2016). We did not find detectable levels of V-ATPase at the plasma membrane of PC-3 cells using the anti-V_1_A antibody (Figs 1B and 4), even though this antibody recognizes all V-ATPase pumps in a cell. We asked whether trafficking of V-ATPase to the plasma membrane is inducible, particularly if cell motility can cause V-ATPase transfer to the plasma membrane in PCa cells, as shown in breast cancer cells (Cotter et al., 2016). Confluent monolayers of PC-3 cells were ‘wounded’ by introducing a scratch. The cells were fixed (4 h post scratch) and V-ATPase cellular localization visualized in the cells farthest from the wound (non-migrating cells in Fig. 7) versus the cells that had moved to the middle of the wound (migrating cells in Fig. 7). Actin cytoskeleton provides cells with mechanical support for vesicle and cell movement, and we monitored F-actin with phalloidin to ask whether V-ATPase co-localized with F-actin. V-ATPase co-localized with F-actin in vesicles near the leading edge of the migrating cells but not in the non-migrating cells. These results indicate that directed movement to the scratch provoked redistribution of V-ATPase-containing vesicles to the front of migrating cells where they co-localize with actin filaments.

### V-ATPase inhibition prompts F-actin reorganization

**Fig. 8.**
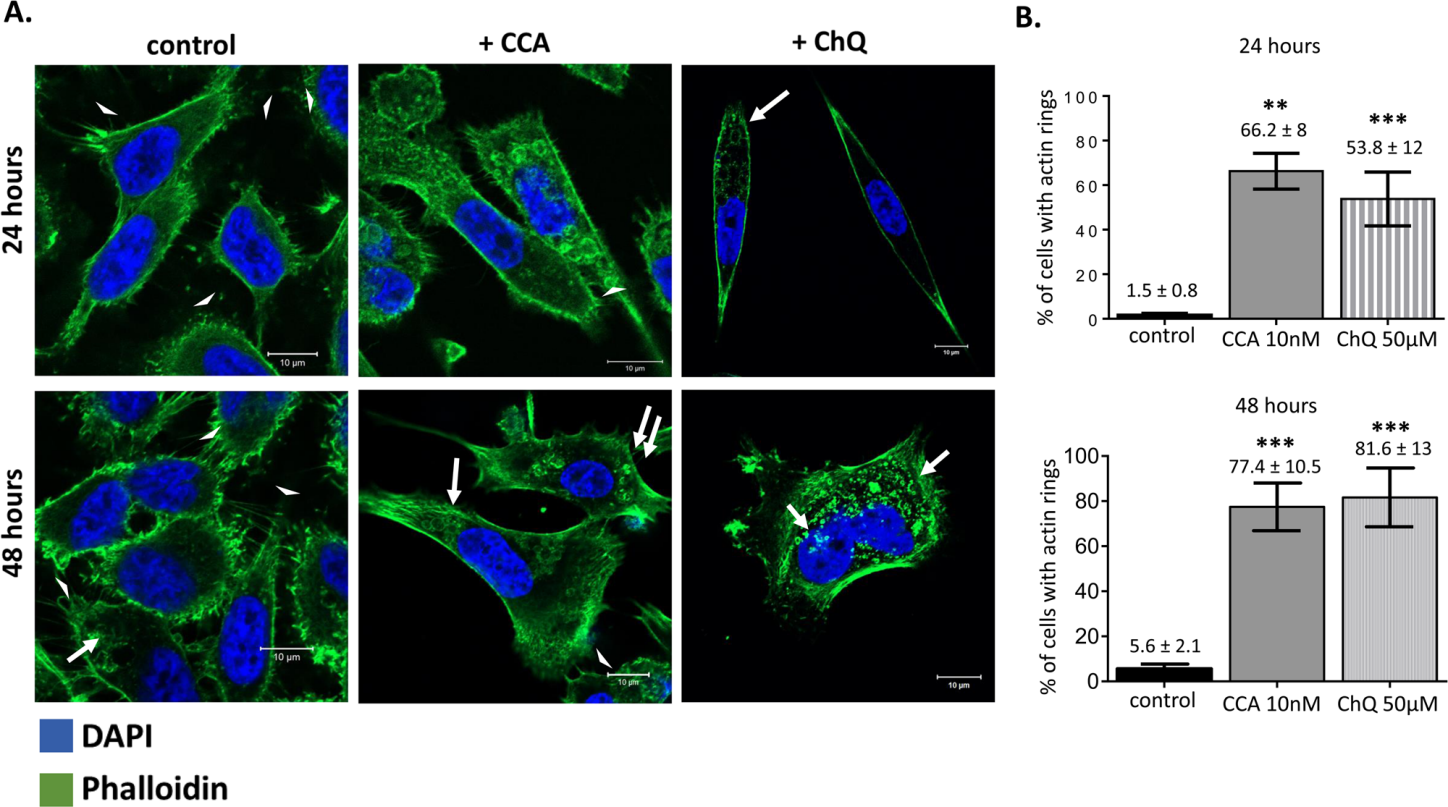
**CCA and ChQ treatment induce accumulation of F-actin rings.** (A) PC-3 cells were fixed after a 24 h (top) or 48 h (bottom) incubation with vehicle control media (DMSO 0.005%), 5 nM of CCA, or 50 µM of ChQ, and then immunostained with phalloidin (green). DAPI (blue) was used as nuclear marker. White arrows show F-actin rings. White arrowheads show filopodial projections. Scale bar: 10 µm. (B) F-actin rings were measured in several microscope pictures and the percentage of cells with F-actin rings counted. *n*≥37 cells, **P<0.01, ***P<0.001, Mann-Whitney test; mean±s.e.m.

We visualized F-actin with phalloidin after V-ATPase inhibition because actin remodeling is pH-dependent and plays important roles in cancer cell motility and invasion (Yonezawa et al., 1985). F-actin bundle filopodial extensions were significantly reduced in CCA-treated cells; these cells exhibited shorter filopodia-like protrusions as compared to untreated cells (arrow heads, Fig. 8A). In addition, F-actin formed ring structures of different sizes after treatment with CCA (arrows, Fig. 8A). The number of cells containing F-actin rings increased from 2% prior to CCA treatment to 66.3±8% after 24 h and 77.5±10.5% after 48 h of CCA exposure (Fig. 8B). Smaller rings also were visible at 48 h. F-actin rings were also observed after treating the cells with chloroquine, which alkalinizes organelles independently of V-ATPase activity. The number of chloroquine- treated cells presenting F-actin rings was nearly the same at both 24 and 48 h. Comparisons between chloroquine- and CCA-treated cells revealed a larger amount of small F-actin rings with chloroquine. These results indicate that disruption of organelle luminal acidic pH and membrane pH gradients with either V-ATPase inhibitors or chloroquine causes F-actin reorganization into rings.

The F-actin rings resembled invadosomes (Linder, 2009) described at the matrix focal degradation area in invasive cancer cells, including PC-3 cells (Artym et al., 2015). However, the adhesion plaque protein vinculin and the protein tyrosine kinase Src substrate Tks5, which are linked to the formation and function of invadosomes, did not co-localize with the rings by immunocytochemistry (Fig. S1). Organelle acidification is also necessary for matrix metalloproteinases transport to the invadosomes and extracellular matrix degradation (Smith et al., 2016; Linder, 2009). However, endo-lysosomal acidification was impaired by V-ATPase inhibitors and chloroquine treatment (Fig. 3C), further suggesting that these F-actin rings were not functional invadopodia. Similar F-actin ring structures assemble on *Dictyostelium discoideum* lysosomes to promote exocytosis of indigestible material (Carnell et al., 2011). We examined the lysosomal markers LAMP1 and LAMP2 to determine whether the F-actin rings resulted from lysosomal V-ATPase retrieval/recycling (Carnell et al., 2011). LAMP1 and LAMP2 were not detected (Fig. S2).

## DISCUSSION

A repertoire of studies have shown that V-ATPase pumps play essential roles in carcinogenesis (Capecci and Forgac, 2013; Cotter et al., 2015; Gerweck et al., 2006; Hinton et al., 2009; Michel et al., 2013; Milito et al., 2007; Montcourrier et al., 1997; Sennoune et al., 2004; Smith et al., 2016; von Schwarzenberg et al., 2014; You et al., 2009). V-ATPase proton transport and its central roles in pH homeostasis contribute to several cellular processes and cancer phenotypes, including invasion and metastasis (Capecci and Forgac, 2013; Cotter et al., 2015; Gerweck et al., 2006; Hinton et al., 2009; Michel et al., 2013; Milito et al., 2007; Montcourrier et al., 1997; Sennoune et al., 2004; Smith et al., 2016; von Schwarzenberg et al., 2014; You et al., 2009). However, the scope of cellular responses driven by V-ATPase inhibition is complex in cancer cells and the mechanisms are poorly understood.

Plasma membrane V-ATPase has been shown to generate a low extracellular pH that is important for the activation of proteases that degrade the extracellular matrix, thereby allowing for metastasis in aggressive cancer cell lines (Appelqvist et al., 2013; Forgac, 2007; Hinton et al., 2009; Jiang et al., 2001; Sennoune et al., 2004). Given that PC-3 cells display highly invasive and androgen-insensitive phenotypes, and that the cell line is considered a good cell model of ablation-resistant prostate cancer (Kaighn et al., 1979; Sobel and Sadar, 2005a,b) and prostatic small cell carcinoma (Tai et al., 2011; Tanaka et al., 2001), it is reasonable to assume that V-ATPase may be present at the plasma membrane of PC-3 cells. Indeed, V-ATPase-dependent proton efflux and extracellular acidification have been previously measured in PC-3 cells (Smith et al., 2016). However, in the present study, V-ATPase subunit V_1_A was negligible on the plasma membrane by immunocytochemistry analyses (Fig. 7). We propose that rather than a direct effect of plasma membrane V-ATPase, downstream extracellular pH alterations may result from changes in cytoplasmic homeostasis when intracellular V-ATPase is not functional. Since V-ATPase is remarkably more abundant intracellularly than on the cell surface (Figs 4 and 7), it is likely that the intracellular pumps are largely responsible for the *in vitro* invasion and cell migration defects induced with BAA and CCA in PC-3 cells.

V-ATPase V_o_a2 and V_o_a3 subunit isoforms are the major V_o_a isoforms expressed in PC-3 cells (Fig. 1A), suggesting that V-ATPase complexes containing V_o_a2 or V_o_a3 are the primary contributors to PC-3 cell invasiveness and migration, intracellular membrane trafficking, and F-actin reorganization into rings. Subunit V_o_a3 is expressed on the plasma membrane of invasive breast cancer cells, pancreatic cells, melanoma cells, and ovarian cancer tissue and has been linked to enhanced tumor cell invasion (Cotter et al., 2016). Interestingly, plasma membrane V-ATPase containing the V_o_a1 subunit isoform was previously shown to contribute to cell invasion in PC-3 (Smith et al., 2016). However, the V_o_a1 subunit isoform was not measurable in our studies, and V-ATPase was not detected on the plasma membrane (Figs 1A and 7). Our results are consistent with a previous report that V_o_a1 transcripts are absent in the PC-3 cells (Liu et al., 2008). One possible explanation to these disparate results is that V_o_a1 expression is inducible under different growth conditions. These results may also simply be a consequence of the methods used to assess V_o_a isoforms (SNP arrays, microarray, qRT-PCR, and western blots). Nonetheless, it remains a challenge to establish how V-ATPase subunit isoforms contribute to specific tumorigenic phenotypes, because subunit isoforms can functionally compensate for each other (Toei et al., 2010), and the V-ATPase inhibitors available do not discriminate between isoforms.

V-ATPase is predominant in the Golgi compartment of PC-3 cells (Fig. 1B,C) and other prostate cancer cells (Michel et al., 2013). In addition, V-ATPase is highly abundant in clathrin-containing cytosolic vesicles (Fig. 1B,C). Its prominent co-localization with giantin and clathrin suggests that V-ATPase activity supports membrane trafficking from the Golgi compartment and cargo trafficking via clathrin-mediated endocytosis, consistent with its crucial roles controlling the intralumenal pH. A large number of cytosolic vesicles accumulated in PC-3 cells treated with the V-ATPase inhibitors (Figs 3C and 4). V-ATPase inhibition leads to cytoplasmic clathrin-coated vesicles buildup (Fig. 4), indicating that the internalized membranes are trafficked into endosomes, but cannot be sorted back to the surface of the cell or into other compartments (lysosomes) for cargo degradation. A substantial increase of Pearson's values in the trans-Golgi network (giantin) and recycling endosome (transferrin receptor), but not in lysosomes (LAMP-1) after V-ATPase inhibition (Fig. 5) is strong evidence of a defective clathrin-independent endocytosis recycling pathway, as well. Accordingly, transferrin receptor is detected in the cytoplasm but not the plasma membrane after V-ATPase inhibition (Fig. 4), suggesting that it is unable to recycle. Although the precise mechanism responsible for defective plasma membrane recycling is not determined in PC-3 cells, V-ATPase inhibition prevents cholesterol from recycling from endosomes back to the plasma membrane in HeLa, retinal pigment epithelial (RPE), and A431 cells depleted of V-ATPase activity (Kozik et al., 2013). Thus, it is conceivable that aberrant plasma membrane cholesterol composition plays a role in PC-3 cells inability to recycle transferrin receptor to the plasma membrane.

The recycling pathway of post-internalized vesicles consists of three main routes for cargo sorting from the early endosome: retrograde traffic (to the trans-Golgi network and back to the plasma membrane), slow traffic (to the recycling endosome and recycled back to the plasma membrane), and rapid traffic (directly back to the plasma membrane) (McDermott and Kim, 2015). Cargo can also be targeted to the lysosomes for degradation. This study suggests that V-ATPase's foremost contribution is to the retrograde and slow traffic recycling pathways rather than the degradation pathway. This study also suggests that retrograde and slow traffic are major recycling routes in the PC-3 PCa cells, and that V-ATPase activity is crucial to deliver vesicles from the Golgi and recycling endosomes to the plasma membrane. Clearly, V-ATPase inhibition leads to widespread vesicle trafficking defects that likely hinder the invasive phenotypes typical of PC-3 cells (Fig. 6). Notably, endocytosis controls internalization of many recep­tors with roles in cellular homeostasis, growth control, and cell differentiation.

In addition to impairing fundamental V-ATPase functions such as endo-lysosomal lumen acidification and vesicle trafficking, cell migration is severely inhibited and prominent F-actin cytoskeleton rearrangements occur when V-ATPase is not active. Cell migration requires F-actin assembly into thin, fingerlike extensions called filopodium that are necessary for motility; interestingly, these projections require intracellular trafficking. (Capecci and Forgac, 2013; Cotter et al., 2016; Michel et al., 2013; Montcourrier et al., 1997). Reduction or elimination of filopodia after treatment with CCA (Fig. 8, arrowheads) indicates that V-ATPase inhibition reduces the migration velocity of the PC-3 cells by disturbing filopodium assembly (Fig. 6). Consequently, V-ATPase offers a therapeutic target for disturbing the integrity of the actin cytoskeleton and prostate cancer progression.

There are multiple actin-based functions known during exocytosis (Meunier and Gutiérrez, 2016; Nightingale et al., 2012). For example, actin rings associated with vesicle membranes provide a stronger force to overcome environmental stress factors during the exocytosis of large granules (Nightingale et al., 2012). In PC-3 cells, the amount of cytoplasmic F-actin rings formed after treatment with sub-lethal concentrations of V-ATPase inhibitors increases from 24 to 48 h (Fig. 8B). We detected similar ring arrangements using anti-transferrin receptor antibodies in PC-3 cells treated with CCA (Fig. 4A, white arrows), suggesting that these F-actin ring structures directly assemble on endosomal membranes. Given our findings that V-ATPase inhibitors lead to an accumulation of endosomes (Fig. 4), we propose that F-actin rings formed in response to V-ATPase inhibition act at the stage of late endosomal recycling to facilitate exocytosis of material trapped within congested endosomes, in an attempt to alleviate traffic defects. A similar function was proposed for WASH–F-actin patches in *Drosophila* (Nagel et al., 2017), although *Drosophila* F-actin ring structures differ from those in PC-3 cells as the *Drosophila* rings retain a lumenal acidic pH. Notably, in the *D. discoideum* amoeba, V-ATPase inhibition or treatment with chloroquine induces assembly of F-actin into rings on the surface of large lysosomes to promote exocytosis of indigestible material (Carnell et al., 2011). However, F-actin co-localization with the LAMP1 and LAMP2 lysosomal markers is negligible in PC-3 (Fig. S2), indicating that assembly of F-actin rings in the PC-3 cells is a distinctive pro-survival response to alleviate traffic defects upon V-ATPase inhibition.

V-ATPase is directly involved in interactions with the actin cytoskeleton (Feng et al., 2009, 2014; Vitavska et al., 2005; Zuo et al., 2008). Actin organization defects resulting from V-ATPase inhibition have been shown in yeast (Drory and Nelson, 2006; Zhang et al., 1998), insects (Vitavska et al., 2005; Wieczorek et al., 2009), and HeLa cells (Kazami et al., 2014). In PC-3 cells, this study shows evidence that proximity of V-ATPase to F-actin is inducible and stimulated by polarized cell migration. V-ATPase co-localizes with F-actin only in vesicles neighboring the leading edge (Fig. 7), not at the plasma membrane as reported in breast cancer cells (Cotter et al., 2016). Thus, spatial-temporal interactions between F-actin and V-ATPase could be cancer- and cell type-specific. For example, V-ATPase does not co-colocalize with actin in the Golgi of PC-3 cells, as reported before in HeLa cells (Serra-Peinado et al., 2016).

In summary, V-ATPases are primarily located in the Golgi compartment and clathrin-coated vesicles in PC-3 cells. The PC-3 cells treated with V-ATPase inhibitors display impaired endo-lysosomal pH, vesicle trafficking defects, compromised migration and invasion, and prominent F-actin reorganization. The finding that PC-3 cells accumulate intracellular F-actin rings that resemble exocytic F-actin rings in response to organelle pH alterations is novel. To our knowledge, V-ATPase-dependent F-actin ring formation has not been described in PCa or any other cancer cell type. Thus, particular F-actin reorganization driven by V-ATPase inhibition may be cancer- and cell type-specific. We propose that these F-actin rings assemble on the surface of organelle membranes to promote their traffic and/or release their contents, as a means of overcoming a widespread vesicle traffic jam caused by organelle pH alterations upon V-ATPase inhibition. Future studies will determine the specific function of these F-actin rings and whether they provide actin-based force to promote exocytosis and reduce toxic accumulation of vesicles and their cargo upon V-ATPase inhibition in PCa cells and other cancers.

## MATERIALS AND METHODS

### Cell culture

PC-3 cells were cultured in RPMI-1640 media (Gibco, Grand Island, NY, USA) supplemented with 10% fetal bovine serum (FBS; SIGMA, St. Louis, MO, USA). All experiments were performed with cells with less than 50 passages and with three biologically independent experiments unless otherwise stated. PC-3 cells were authenticated using short tandem repeat profiling and were free of mycoplasma contamination.

### Quantitative real-time PCR (qRT-PCR)

RNA was isolated using the RNeasy Mini kit (Qiagen, Germantown, MD, USA) and reverse transcribed with the RETROscript^®^ cDNA kit (Applied Biosystems, Foster City, CA, USA). Primers were designed using the PrimerQuest tool from Integrated DNA Technology. The following forward (*f*) and reverse (*r*) primers were used: GUSB (*f*: CTCATTTGGAATTTTGCCGATT; *r*: CCGAGTGAAGATCCCCTTTTTA), V_1_A (*f*: GCCCATTCTACAAGACAGTAGG; *r*: CTCCCATGTGCTCACGAATAA), V_o_a1 (*f*: CACTGGGTTGAGTTCCAGAATA; *r*: TCACTCTTCAAACTTCCCTTCC), V_o_a2 (*f*: TCTGTCCCTGTCCTCTTCTT; *r*: CCTTATAAGTGTGTAGCCACTCC), V_o_a3 (*f*: ATGACCTTCCTCATCTCCTACT; *r*: GCTGCAGAAACGGGAAGA), V_o_a4 (*f*: TGATTTCTGTGCCGTGGATG; *r*: TGTTCTCAGTGGCATCTTCTTG). qRT-PCR was performed with SYBR Green I Mastermix (Roche, Indianapolis, IN, USA) on a Roche LightCycler 480 II. Analysis was performed using ΔΔC_t_ method and expression of β-glucuronidase (GUSB) was used to normalize for variances in cDNA input (Fordyce et al., 2012). Samples were analyzed in four independent experiments.

### Cell viability assay

Cell viability was assessed with Tetrazolium MTT [3-(4, 5-dimethylthiazolyl-2)-2, 5-diphenyltetrazolium bromide] Assays (ATCC, Manassas, VA, USA). The cells were exposed to two different V-ATPase inhibitors, bafilomycin A (BAA) (VWR, Radnor, PA, USA) or concanamycin A (CCA) (Wako, Japan), at the indicated doses and times. Viable cells reduce MTT, which is measured by absorbance. Data was expressed as optical density (OD) relative to the vehicle-treated control.

### Acridine Orange staining

To assess changes in pH of acidic vesicles, cells were incubated with Acridine Orange (SIGMA, St. Louis, MO, USA; 1 µM in media) for 30 min at 37°C, then fixed on glass slides with 4% paraformaldehyde. Slides were imaged with META/AxioObserver (Thornwood, NY, USA).

### Endosome/Lysosome pH measurements

Cells were incubated with 1 mM 8-hydroxypyrene-1,3,6-trisulfonic acid (HPTS) (Life Technologies, Carlsbad, CA, USA) for 16 h, then treated with vehicle (0.005% DMSO), 5 nM CCA (Wako, Japan), or 50 µM chloroquine (SIGMA, St. Louis, MO, USA) for 1 h. Fluorescence was measured using a FluoroMax 4 spectrofluorometer (Horiba Jobin Yvon, Irvine, CA, USA) with an excitation ratio of 458/405 nm at a fixed emission of 515 nm. The HPTS fluorescence excitation 458/405 ratio was determined and converted to pH values by comparison to standard curves generated using known pH buffers (i.e. pH 5 to pH 8) using a non-linear regression.

### Immunocytochemistry

Immunocytochemistry was performed at room temperature following standard procedures (Michel et al., 2013). Line profiles of fluorescent intensity were obtained using ZEN 2009 Light Edition © Carl Zeiss MicroImaging software. Pearson's correlation *r* values were used to characterize the degree of overlap between fluorescent channels, using SlideBook 5.0 software (www.intelligent-imaging.com/slidebook). The V-ATPase subunit V_1_A antibody was generated by BioGenes (Berlin, Germany) and validated (Michel et al., 2013). The antibodies to LAMP1 (lysosome marker, ab25630), giantin (Golgi marker, ab37266) and clathrin (vesicle marker, ab2731) were purchased from Abcam (Cambridge, UK). The antibody for Tsk5 (invadosome marker, sc-376211) was purchased from Santa Cruz Biotechnology (Dallas, TX, USA). The antibody rodamine-phalloidin (R415) was obtained from Thermo Fisher (Waltham, MA, USA). The antibody to transferrin receptor (endocytic vesicle marker, 136800), the antibody for AlexaFluor488-phalloidin, and the secondary antibodies AlexaFluor488 (A-11001) and AlexaFluor546 (A-11010) were purchased from Invitrogen (Grand Island, NY, USA).

### Motility and invasion assays

*In vitro* motility and invasion assays were performed following manufacturer's protocols (BD Biosciences, San Jose, CA, USA) in 24-well plates containing 2.5×10^4^ cells plated on control or matrigel-coated inserts. Cells were treated with BAA or CCA, 5 nM, or vehicle (0.005% DMSO) for 24 h. FBS (10%) was used as the chemoattractant. Invaded cells were fixed and stained and then counted using a microscope (10×, ZEISS Axiovert 25).

### Western blot

RIPA buffer was utilized to prepare whole cell lysates using standard procedures (Michel et al., 2013). Protein concentrations of whole cell lysates were determined using BCA assay (Pierce), and 100 µg of protein were diluted in 4× Laemmli Buffer prior to loading on 8% polyacrylamide gels. Primary antibodies against V_1_A (Michel et al., 2013) and β-actin (SIGMA) were diluted in 5% milk in TBS-T 1:1000. Immunoblots were imaged using the ChemiDocTM XRS workstation (BioRad).

### Wound healing assay

A confluent monolayer of cells was ‘scratched’ with a 200 µl pipette tip to create a ‘wound’ to induce motility in the absence (0.005% DMSO) or presence of BAA or CCA, 5 nM. Scratch width (µm) was imaged every 2 h using a 20× objective. For analysis, scratch width (µm) was determined using AxioVision LE (Carl Zeiss Microscopy) software. Scratch width was compared between conditions at each time point.

### Statistical analysis

Mann–Whitney tests were performed to determine statistical significance between groups using GraphPad Prism 5 software (San Diego, CA, USA). *P*<0.05 was considered statistically significant. Pearson's correlation values (Pearson's *r*) were obtained to characterize co-localization using the degree of overlap between the green fluorescent channel (protein markers: clathrin, transferrin receptor, giantin, and LAMP1) and the red fluorescent channel (V-ATPase subunit V_1_A); images were analyzed using SlideBook 5.0 software. Pearson data is either presented as *r* values (to assess co-localization; Fig. 1), or normalized to control (to compare changes in co-localization between control versus V-ATPase inhibitor-treated groups; Fig. 5).

## Supplementary Material

Supplementary information
