# Supplementary material for: F-actin reorganization by V-ATPase inhibition in prostate cancer
Source: Biol Open. 2017 Oct 16;6(11):1734–44. doi: 10.1242/bio.028837 (PMC5703614; doi:10.1242/bio.028837)
Supplement: Supplementary information [file biolopen-6-028837-s1.pdf]

SUPPLEMENTAL FIGURES

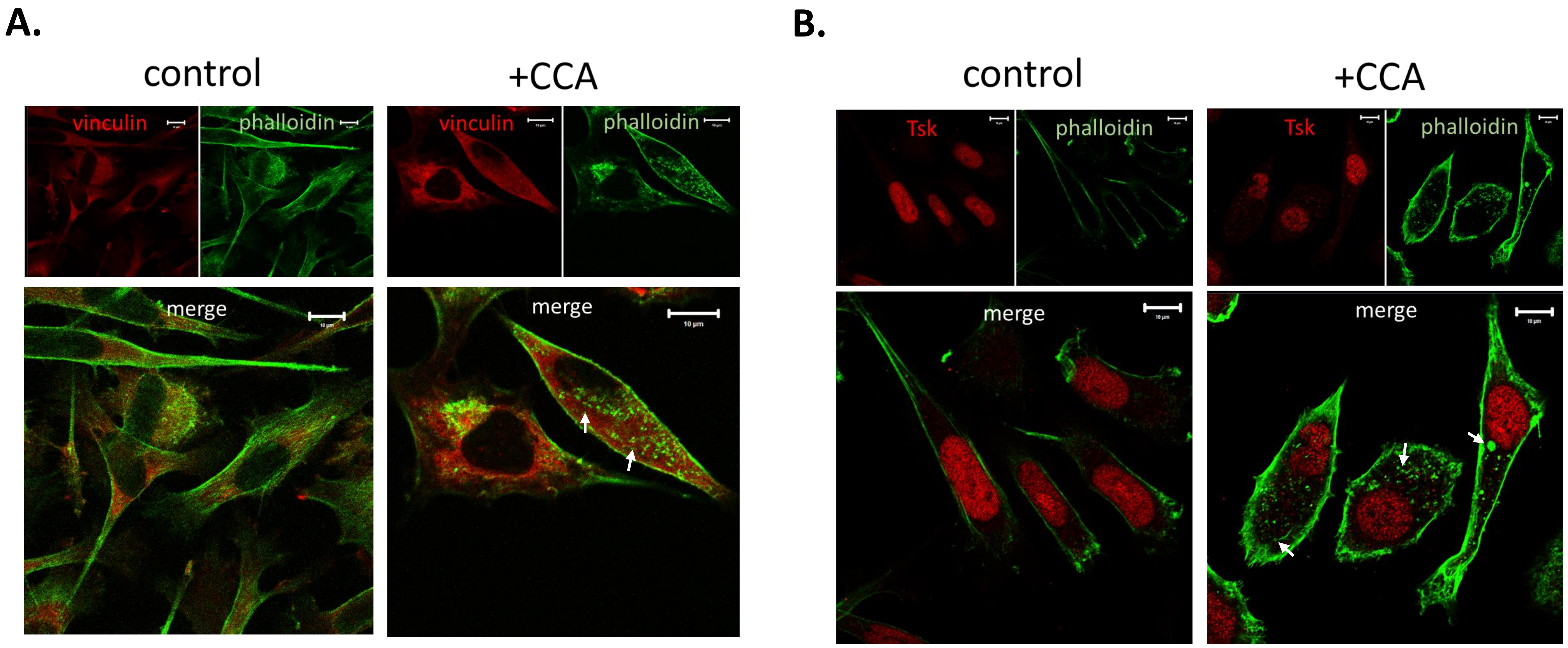

FIGURE S1

FIGURE S1. CCA-induced F-actin rings do not co-localize with vinculin or Tsk5. PC-3 cells were incubated with vehicle control media (DMSO 0.005%) or V-ATPase inhibitor (+ CCA) for 48 hours and then fixed and co-immunostained with antibodies against the invadosome markers vinculin (**A**, red) or Tsk5 (**B**, red) and phalloidin (F- actin marker, green). White arrows = F-actin rings. Scale bars = 10 *μ*m.

Biology Open • Supplementary information

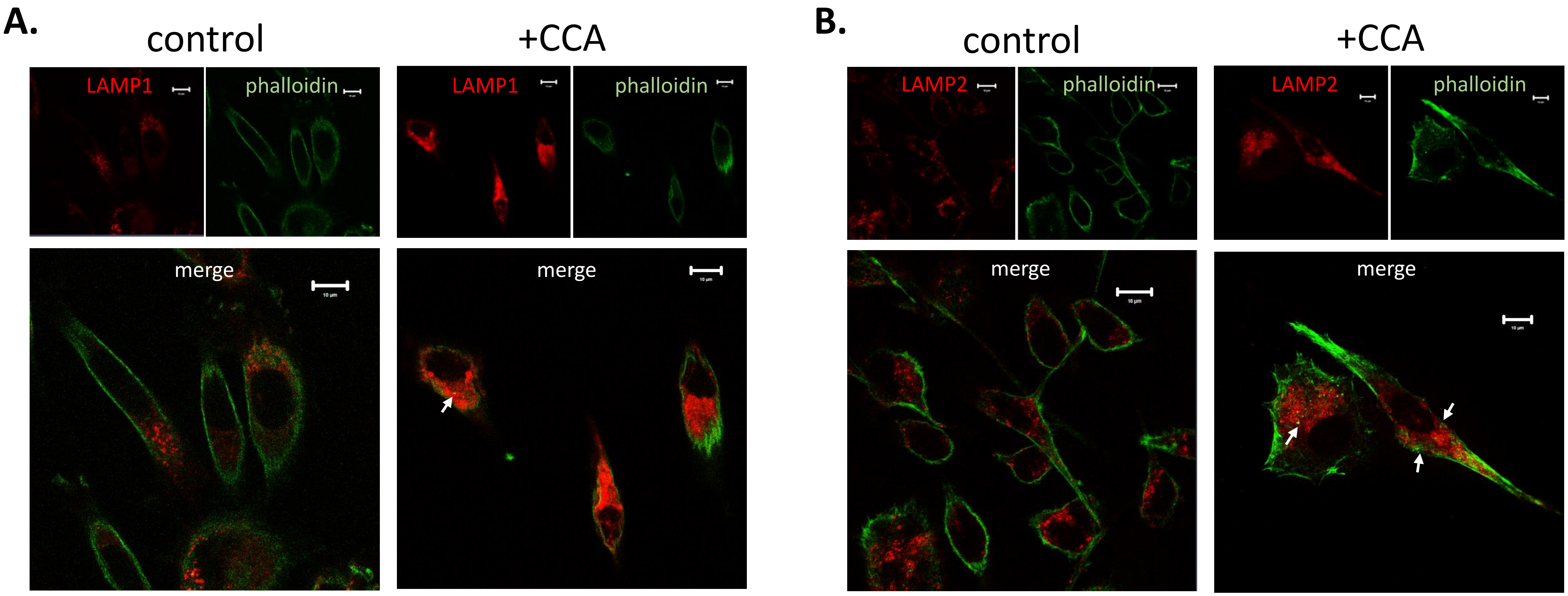

FIGURE S2

FIGURE S2. CCA-induced F-actin rings do not co-localize with LAMP1 and LAMP2. PC-3 cells were incubated with vehicle control media (DMSO 0.005%) or V-ATPase inhibitor (+ CCA) for 48 hours and then fixed and co-immunostained with antibodies against the lysosome markers LAMP1 (**A**, red) or LAMP2 (**B**, red) and phalloidin (F-actin marker, green). White arrows = F-actin rings. Scale bars = 10 μm.

Biology Open • Supplementary information
